# Is it safe to remove the chest tube in the operating room after robotic lobectomy, segmentectomy, and wedge resection with lymphadenectomy?

**DOI:** 10.3389/fsurg.2025.1719281

**Published:** 2025-12-04

**Authors:** Ashley J. McCormack, Katherine G. Phillips, Robert J. Cerfolio

**Affiliations:** Department of Cardiothoracic Surgery, NYU Langone Health, New York, NY, United States

**Keywords:** chest tubes, robotic pulmonary resection, lymphadenectomy, process improvement, prospective

## Abstract

**Background:**

We have previously shown that it is safe to remove chest tubes within four hours after robotic pulmonary resection with aggressive thoracic lymphadenectomy in patients without an air leak.

**Methods:**

This is a prospective quality improvement study that examines the process of removing chest tubes before the patient leaves the operating room (OR) after robotic pulmonary resection. Chest tubes were removed in the OR if the air leak was ≤75 mL/min on a digital drainage system. The tubes were reinserted only for oxygen desaturations from increasing pneumothorax and/or increasing subcutaneous emphysema.

**Results:**

Between 1 March 2023 and 12 December 2024, 223 consecutive patients underwent pulmonary resection with complete lymphadenectomy by one surgeon. Overall, 130 patients (58%) had their chest tubes removed in the OR, in 54% (62/114) of lobectomies, 62% (48/78) of segmentectomies, and 65% (20/31) of wedge resections. Thirteen patients (10%) required chest tube reinsertion, 11 after lobectomy and 2 after segmentectomy. The median operative time was 90 min (range 29–244 min), blood loss was 20 mL (range 10–60 mL), and all but one patient went home on postoperative day 1. No 30-day or 90-day mortality rate was recorded. A postoperative thoracentesis was performed in 1% of patients.

**Conclusion:**

Chest tubes can be safely removed in selective patients before they leave the OR after a robotic pulmonary resection with complete lymphadenectomy. Factors that may lead to these outcomes are the meticulous intraoperative technique and hemo-chylostasis. An air leak threshold of <20 mL/min may be optimal to minimize chest tube reinsertions and reduce failure rates.

## Introduction

Chest tubes and air leaks remain the most common problem among patients and complaints from surgeons following lung surgery. Thoracic surgeons were early developers of the concept of fast-tracking pulmonary resection (1), later called enhanced recovery (ERAS) pathways (2), yet we can do better in this area. Chest tubes hurt patients, including even the 20-French soft tubes that we use today. In addition, they add significant cost, workforce utilization, and confusion regarding how to manage them. Therefore, the best way to manage chest tubes is to eliminate them like it is done for complications.

We have previously shown in 2023 that it is safe to avoid chest tubes after a robotic thymectomy (3), and others have shown the safety of chest tube removal after thoracoscopic wedge resections in the operating room (OR) (4). We have reported on over 300 patients between 2020 and 2023 that it is safe to remove chest tubes in the recovery room within 4 h after pulmonary lobectomy, segmentectomy, and wedge resection even after an aggressive complete thoracic lymphadenectomy that averages five N2 and three N1 stations with a median of 27 total lymph nodes removed, provided there is no air leak or chylous fluid (5, 6). In that series, patients were given ice cream by mouth in the recovery room prior to chest tube removal as a provocative test for chylothorax. The goal of this study is to assess the safety of chest tube removal before patients leave the OR after a robotic pulmonary resection with complete thoracic lymphadenectomy and to eliminate tubes altogether, from the postoperative period, surgical discourse, and algorithms.

## Patients and methods

This is a prospective quality improvement study that examines the safety of removing chest tubes before the patient leaves the OR on a consecutive series of patients. All patients who underwent robotic pulmonary wedge resection, segmentectomy, or lobectomy with complete thoracic lymphadenectomy by one surgeon (RC) from 1 March 2023 to 12 December 2024 were included in this study. There were no contraindications to a robotic platform (no matter the tumor size) nor were there attempts to remove the chest tube in the OR. All perioperative data were retrospectively collected and reviewed. Major and minor adverse events, complications, and readmissions within 30 days of the operation were included in the perioperative data. The study design, including a waiver of patient consent, was approved by the Institutional Review Board at NYU Langone Health #i24-01163.

Each pulmonary resection was conducted using the da Vinci Xi Surgical System (Intuitive Surgical, Sunnyvale, CA, USA) through a portal four-arm approach and an additional assist port, as reported by us previously (7, 8). At the conclusion of the operation, a single 20-Fr chest tube was inserted in the access port, positioned posteriorly and apically, connected to a digital drainage system, called the Thoraguard Centese system (Omaha, NE, USA), and set to −20 cmH_2_O suction (9). The remaining part of the lung was observed to inflate to ensure that there was no atelectasis or torsion.

The digital device was not powered on until the deep layers of the small incisions of the extraction site had been closed to prevent ambient air from entering the chest. As the subdermal and dermal layers of the portal incisions were closed with a subcuticular knotless technique, the air leak was continuously assessed on the air leak monitor. The decision to remove the chest tube was made at three different time points in the OR:

1. The first was at the end of the operation with the patient still intubated and on their side. If the air leak was continuously 75 mL/min or less, the tube was removed. This was performed by asking the anesthesiologist to deliver a Valsalva maneuver as the chest tube was quickly removed. The chest tube site was then closed with one deep interrupted suture and a knotless subcuticular suture on the skin. Normal ventilations were resumed by the anesthesiologist.
2. If the tube was not removed in Step 1 at the end of the operation with the patient still intubated in the lateral decubitus position and the air leak was greater than 75 mL/min, we placed one deep suture and threaded a subcuticular closure around the tube and turned the patient supine. If the leak was 75 mL/min or less after repositioning the patient supine, then we removed the tube as described above as the patient was being extubated.
3. Finally, if the tube still remained inside after Steps 1 and 2, the air leak was assessed again. After the patient was transferred to the bed from the OR table and just prior to wheeling the patient out of the room, the air leak was assessed a third time. If the leak was 75 mL/min or less, the tube was removed as described above.

A chest X-ray (CXR) was performed in the recovery room on all patients. If a pneumothorax was present on the CXR, the height of the pneumothorax from the apex was measured in millimeter. Chest tubes were reinserted only for oxygen desaturations associated with increasing pneumothorax and/or increasing subcutaneous emphysema. They were not reinserted on the basis of CXR alone if a patient was stable, asymptomatic, and had adequate oxygenation saturations irrespective of the size of the pneumothorax. If chest tube reinsertion was required, it was performed at the bedside using standard sterile procedures with either a 14-Fr pigtail or a small-bore chest tube inserted through one of the robotic incisions used earlier. Most commonly, the camera port was used for chest tube reinsertion. A CXR was obtained after chest tube reinsertion to confirm placement.

The chest tubes were connected to a digital drainage system and then removed once the air leak was ≤30 mL/min with a negative pleural assessment (only negative numbers when the patient is asked to breathe in and out deeply) after the patient was out of the OR. The tubes were removed according to these guidelines by the members of our surgical team, who include advanced practice providers and house staff, regardless of the time of day or hour after the operation and assessed every time rounding was performed. Ongoing air leaks without pneumothorax were treated with a water seal. If the chest tubes were not removed in the OR, then our previously published postoperative chest tube care algorithm was used, which involves removing chest tubes any time postoperatively after ice cream is consumed in the recovery room (5, 6, 10). If there was an ongoing air leak, as defined by a positive pleural assessment or an air leak of ≥30 mL/min on the morning of postoperative day 1 (POD1), patients were discharged home at 8 AM with their chest tubes in place as described by us previously (10).

The patients were prescribed cephalexin 250 mg only once a day by mouth. They were also instructed to send daily texts and/or videos to the attending surgeon of the air leak reading, home pulse oximetry reading, and how they were feeling.

## Results

Between 1 March 2023 and 12 December 2024, 223 patients underwent a robotic pulmonary resection with complete thoracic lymphadenectomy. All patients seen during this time were offered a robotic platform and all were included in the study. Demographics of the cohort are given in Table 1. Of the 223 patients, 114 patients underwent lobectomy (51%), 78 underwent segmentectomy (35%), and 31 underwent a wedge resection (14%), as shown in Table 2.

**Table 1:** Demographics and patient characteristics of the overall cohort, of patients whose chest tubes were removed in the OR, and of patients whose chest tubes remained in place in the OR.

Variable	Consecutive series of patients in the study *N* = 223	Patients with chest tubes removed before leaving the OR *N* = 130	Patients with chest tubes remaining after exiting the OR *N* = 93
Age, years, median (range)	70 (13–95)	68 (18–82)	73 (13–95)
Sex, *n* (%)			
Female	125 (56%)	73 (56%)	52 (56%)
Male	98 (44%)	57 (44%)	41 (44%)
Neoadjuvant treatment, *n* (%)	22 (10%)	11 (8%)	11 (12%)
FEV_1_%, median (range)	96 (45–144)	98 (51–144)	90.5 (45–133)
D_LCO_ %, median (range)	82 (30–126)	83 (30–126)	78.5 (32–120)
Eastern Cooperative Oncology Group (ECOG) score, *n* (%)			
0	132 (65%)	85 (72%)	47 (56%)
1	68 (33%)	32 (27%)	36 (43%)
2	2 (1%)	1 (0.8%)	1 (1%)
3	2 (1%)	2 (1.7%)	
Diabetes mellitus, *n* (%)	27 (12%)	13 (10%)	14 (15%)
Coronary artery disease, *n* (%)	35 (16%)	17 (13%)	18 (19%)
COPD, *n* (%)	12 (5%)	6 (5%)	6 (6%)
Hypertension, *n* (%)	123 (55%)	67 (52%)	56 (60%)
Hyperlipidemia, *n* (%)	121 (54%)	63 (48%)	58 (62%)

FEV_1_, forced expiratory volume in 1 s; D_LCO_, diffusing capacity of the lungs for carbon monoxide; COPD, chronic obstructive pulmonary disease.

**Table 2:** Operative details.

Variable	Consecutive series of patients in the study *N* = 223	Patients with chest tubes removed before leaving the OR *N* = 130	Patients with chest tubes remaining after exiting the OR *N* = 93
Wedge resection, *n* (%)	31 (14%)	20 (15%)	11 (12%)
Segmentectomy, *n* (%)	78 (35%)	48 (37%)	30 (32%)
Lobectomy, *n* (%)	114 (51%)	62 (48%)	52 (56%)
Estimated blood loss in cc, median (range)	20 (10–60)	20 (10–60)	20 (10–60)
Operative time in minutes, median (range)	90 (29–244)	83 (29–244)	104 (50–237)
R0 resection, *n* (%)	221 (99%)	130 (100%)	91 (98%)
Lymph nodes removed, median (range)	29 (14–81)	30 (14–54)	29 (14–81)

Out of the entire cohort, 130 patients (58%) had their chest tubes removed in the OR. The tubes were removed in 62 of the 114 patients (54%) who underwent lobectomy, 48 of the 78 patients (62%) who underwent segmentectomy, and 20 of the 31 patients (65%) who underwent wedge resection.

Two (0.9%) 30-day readmissions were reported (Table 3). One case pertained to a patient whose chest tube was removed in the OR and suffered a stroke on POD7 following lobectomy. The second case was a patient who developed an acute on chronic kidney injury and atrial fibrillation and confusion; this patient's chest tube was not removed in the OR. No 30- or 90-day mortality was reported.

**Table 3:** Postoperative complications.

Variable	Consecutive series of patients in the study *N* = 223	Patients with chest tubes removed before leaving the OR *N* = 130	Patients with chest tubes remaining after exiting the OR *N* = 93
Postoperative thoracentesis, *n* (%)	2 (0.9%)	2 (1.5%)	0
30-day readmission, *n* (%)	2 (0.9%)	1 (0.8%)	1 (1%)
30-day mortality, *n* (%)	0	0	0
90-day mortality, *n* (%)	0	0	0

Of the 130 patients who had chest tubes removed in the OR, a CXR in the recovery room showed a pneumothorax in 58 patients (45%) with a median apical size of 22 mm (range 5–54 mm). Of the 93 patients who had a chest tube in place at the end of the operation, the CXR showed a pneumothorax in 51 patients (55%) with a median apical size of 15 mm (range 1–31 mm).

Thirteen out of the 130 patients (10%) whose chest tubes were removed in the OR required a chest tube reinsertion within 6 h after they demonstrated symptoms such as shortness of breath, increasing subcutaneous emphysema, and/or oxygen saturations below their baseline (Table 4). These included two patients after they underwent segmentectomy and 11 patients after they underwent lobectomy. Of these 13 patients, three went home with a chest tube in place on POD1. The remaining 10 patients had their chest tubes removed before discharge on the morning of POD1.

**Table 4:** Patient characteristics and operative details of patients who required chest tube reinsertion as compared to those who did not.

Variables	Required a new chest tube to be replaced after the chest tube was removed in the OR *N* = 13	Did not require a new chest tube to be replaced after the chest tube was removed in the OR *N* = 117
Patient characteristics		
Age, years, median (range)	73 (52–80)	68 (18–82)
Sex, *n* (%)		
Female	7 (54%)	66 (56%)
Male	6 (46%)	51 (44%)
Diabetes mellitus, *n* (%)	1 (8%)	12 (10%)
Smoker, *n* (%)	8 (62%)	53 (45%)
Steroid use, *n* (%)	0	5 (4%)
Reoperation, *n* (%)	1 (8%)	3 (3%)
Wedge resection, *n*	0	20
Right upper		6
Right middle		2
Right lower		4
Left upper		5
Left lower		3
Segmentectomy, *n*	2	46
Right S1		2
Right S2	1	7
Right S3	1	2
Right S1, 2		3
Right S8, 9		1
Right S6		4
Right S10		2
Right S7, 8		1
Left S2		3
Left S1, 2, 3		2
Left S1, 2		3
Left S4, 5		4
Left S3, 4, 5		3
Left S6		2
Left S8		2
Left S8, 9, 10		3
Left S10		2
Lobectomy, n	11	51
Right upper lobectomy	11	18
Right middle lobectomy	6	9
Right lower lobectomy	3	6
Left upper lobectomy	1	5
Left lower lobectomy	0	9
Right upper lobe sleeve	1	4

A further analysis of these 13 patients is provided in Table 4, and in Tables 5, 6, their air leaks are compared with the 117 patients who had undergone successful removal of their chest tubes before leaving the OR. The size of their air leaks at the time of closure, just after the patients were turned supine and just before they left the OR is shown. The median air leak at time of closure was 59 mL/min in the 13 patients who required chest tube reinsertion and only 35 mL/min in the 117 patients who underwent successful removal of their chest tubes in the OR. If we had used an air leak threshold of 30 mL/min at any of the three time points when the air leaks were assessed in the OR, only six patients would have required chest tube reinsertion (Table 7). Furthermore, if the threshold had been lowered to 20 mL/min, only four patients of the entire series would have required chest tube reinsertion. When specifying by type of resection, right upper lobectomy was the most common type of lobectomy performed. It was performed in 6 of the 13 patients who required chest tube reinsertion, although conversely, it was performed in 18 of the 51 lobectomy patients who underwent successful chest tube removal in the OR. Notably, four patients who underwent right upper lobe sleeve resection also had successful removal of their chest tubes in the OR.

**Table 5:** Air leak measurements of the 13 patients who required chest tube reinsertion as compared to the 105 patients who did not.

Variable	Required a new chest tube to be replaced after the chest tube was removed in the OR *N* = 13	Did not require a new chest tube to be replaced after the chest tube was removed in the OR *N* = 117
Air leak at time of closure (mL/min), median (range)		
Wedge resection	n/a	19.5 (5–466)
		(*n* = 20)
Segmentectomy	57.5 (35–80)	17 (0–83)
	(*n* = 2)	(*n* = 46)
Lobectomy	42 (5–89)	35 (5–109)
	(*n* = 11)	(*n* = 51)
Air leak by patient position on the OR table (mL/min), median (range)		
Lateral decubitus	59 (5–1,200)	35 (1–705)
	(*n* = 9)	(*n* = 95)
Supine	33.5 (19*–*42)	23 (5–48)
	(*n* = 4)	(*n* = 22)

**Table 6:** Exact air leak measurements of the 13 patients while in the lateral decubitus and supine positions in the OR.

Patients	Air leak at time of closure while in the lateral decubitus position (mL/min)	Air leak after positioning supine (mL/min)
13 patients whose chest tubes were removed in the OR but needed replacement		
Patient 1	5	
Patient 2	5	
Patient 3	13	
Patient 4	23	
Patient 5	35	
Patient 6	50	
Patient 7	56	
Patient 8	62	
Patient 9	75	
Patient 10	145	75
Patient 11	279	19
Patient 12	1,036	25
Patient 13	1,200	42

**Table 7:** Number of patients who would have required chest tube reinsertion vs. those who would have had successful removal if we had changed the air leak threshold.

Air leak threshold (mL/min)	Number of patients who would have required chest tube reinsertion	Number of patients with successful chest tube removal
10	2	32
20	4	58
30	6	67
40	7	86
50	9	96
60	10	103
70	11	104
80	13	117

Of the total 223 patients, 222 were discharged home on POD1 and one went home on POD2 for social reasons. Discharge was done before 8:30 AM in 65% of patients, before 10:30 AM in 25%, and later in the remaining 10% of patients. Two patients whose chest tubes were removed in the OR after lobectomy underwent a postoperative thoracentesis for shortness of breath and a moderate pleural effusion on POD17 and POD24, respectively. No postoperative chylothorax was reported in the group of patients whose chest tubes were removed in the OR; however, a low-volume chylothorax was diagnosed in a patient whose chest tube remained for plugging an ongoing air leak. This patient was discharged home with a chest tube in place and the chylothorax resolved at home within three days after the patient followed a low to no fat diet.

Of the 93 patients whose chest tubes remained at the conclusion of the operation, 20 (22%) were discharged home with the chest tube in place. All chest tubes in these patients were removed on an outpatient basis either in the general ward or in the emergency department within 12 days postoperatively. The remaining 73 patients had their tubes removed prior to discharge. The tubes were removed within 2–4 h postoperatively in 22 (30%) patients, 4–6 h in 34 (47%) 6–8 h in 9 (12%), and prior to discharge in the remaining 8 (11%) patients.

## Discussion

Chest tubes continue to generate debates for thoracic surgeons and pain for patients. Dogma and doctrine dominate the discourse, while data are extremely limited. The benefits of eliminating chest tubes are numerous, including minimizing postoperative morbidity, pain, and anxiety, all of which reduce patient satisfaction. In addition, it reduces postoperative work for the already busy and stressed thoracic team.

There are several salient findings in this study. First, we have shown that it is safe to remove chest tubes in the OR in 65% of patients who undergo a robotic wedge resection, 62% who undergo robotic segmentectomy, and 54% who undergo robotic lobectomy. Overall, nearly 60% of patients after undergoing a pulmonary resection with aggressive complete thoracic lymphadenectomy can have their chest tube removed. If we had selected an air leak of 20 instead of 75 mL/min, we would have had only four failures as opposed to 13.

Second, only two patients (0.9%) in this series of 223 patients underwent a postoperative thoracentesis despite early tube removal in the OR and irrespective of the amount of chest tube effluent, even though a complete thoracic lymphadenectomy removes nodes from all five N2 stations and at least two N1 stations with a median of 27 total lymph nodes removed: 31 nodes on the right (range 13–61) and 21 nodes on the left (range 11–41). We attribute this outcome to a host of variables. A technical point is that we carefully retract the lung in a non-grasping passive manner to help mitigate remote air leaks and perform our thoracic lymphadenectomy prior to the pulmonary resection and routinely pack stations 2R, 4R, and 7 with a rolled gauze. We then inspect these stations carefully at the end of the operation for any signs of subtle chyle leak or bleeding. Also, we actively participate in the postoperative care of all of our patients, including after discharge, using daily texts and pulse oximetry information, as described by us previously (10, 11), and in the care of even those who travel to our institution by airplane. This mitigates unnecessary procedures done by home physicians. Furthermore, the amount of drainage is irrelevant for chest tube management, as we and others have shown for over a decade now (12). In this study, we have shown that it does not even require inspection since tubes can be removed in many patients in the OR (this study) or within 4 h (previous study) (6).

Third, in this study, we show that 49% of patients (109/223) develop pneumothoraces after pulmonary resection even if they have a chest tube in place (55% of patients with a chest tube and 45% of patients without a chest tube). This space seen on the CXR has little if any clinical value. These data suggest that postoperative CXRs (even the initial CXR in the recovery room) provide little if any meaningful actionable information. More than one-third of CXRs taken immediately in the recovery room on patients without OR chest tubes showed pneumothoraces; however, the majority of these were observed. We did not replace the chest tubes based on imaging criteria alone. We also did not find a correlation between clinical symptoms and air leak value.

Fourth, 13 patients required chest tube reinsertion because of symptoms, which can be considered a failure rate of 10%. The risk of reinsertion was highest after lobectomy, with minimal risk of chest tube reinsertion after segmentectomy or wedge resection. Although none of these patients had major morbidity, we had to reinsert a tube that involved additional work, patient discomfort and anxiety, as well as cost and repeat CXRs. We chose an air leak threshold of 75 mL/min based on our prior experience with the digital drainage system. We have previously seen patients in the recovery room do well if we remove their chest tubes when the air leak is 30 mL/min or less, and therefore, here, we surmised that the air leak threshold should be higher in patients in the OR who were intubated with positive pressure ventilation. If we had selected an air leak of 20 mL/min as the criteria to remove the chest tubes in the OR instead of 75 mL/min, our failure rate would have been reduced, although we would have successfully removed tubes only in 58 patients.

Finally, removing chest tubes intraoperatively bypasses our previously published “ice cream challenge” to provoke a chylothorax in the recovery room (5). There may be a small, unquantified risk of delayed or missed chylothorax in patients whose chest tubes are removed in the OR. Although in this series, no chylothorax was reported in the cohort whose chest tubes were removed in the OR, one case of chylothorax did occur in the group whose chest tubes were removed later.

The strengths of this study are as follows: this study was on a consecutive series of patients with no preselection. All patients who underwent a robotic pulmonary resection were included with the intent to remove their chest tubes in the OR. Only the objective air leak data on the digital drainage system were used and not patient-specific risk factors such as redo surgery, low D_LCO_, upper vs. lower lobe resection, and so on. In addition, as our experience and comfort with the technique grew throughout the study period, we gained valuable insights like it may be preferable to remove chest tubes in the supine position when the air leak is lower as compared to in the lateral decubitus position. For these reasons, we believe that this practice can be adopted by other surgeons who follow the techniques we have described here.

There are several limitations to this study. The exact air leak number on the digital drainage system that is most likely to result in successful chest tube removal in the OR is not yet known. As mentioned previously, the number we chose here was based on our prior experience with the digital drainage system used at our institution, and our results would, of course, have been different if we had selected another threshold (6, 10). We have not corroborated the air leak of the Centese system with the leaks of other commercially available systems or even with those of analog systems. Ongoing studies will allow us to determine the optimal air leak threshold for removal.

The scalability of this technique requires many factors to consider. First, the intraoperative technique must be meticulous to avoid pulmonary parenchymal injury by passive retraction of the lung and also avoid active lung retraction that is characterized by grasping the lobes that are not resected and/or closing instruments on them during lymph node dissection or pulmonary resection. Second, the aim must be that postoperative bleeding never occurs. Third, chyle leaks need to be carefully evaluated by using bipolar energy as well as clips as needed to ensure hemostasis and prevent lymphostasis, particularly in lymph node stations 7 and 2R, as described previously (5). Lymph node resection should be done first and packed so that subtle leaks can be identified as they may take 10–20 min to become visible. Fourth, we use a digital air leak system that eliminates interobserver bias and allows specific, reproducible criteria to be set for chest tube removal. Lastly, and perhaps most importantly, we aim to foster a culture that sparks innovation, challenges dogma, and accepts some failures in continuation of efforts to reduce patient morbidity and improve patient experience. Along these lines, we have now started a new protocol to further refine our processes for removing chest tubes following a robotic wedge resection, lobectomy, and segmentectomy. This work will be published in future studies.

## Data Availability

The raw data supporting the conclusions of this article will be made available by the authors without undue reservation.
